# Evaluation of antimicrobial susceptibility testing of *Nocardia* spp. isolates by broth microdilution with resazurin and spectrophotometry

**DOI:** 10.1186/s12866-021-02394-w

**Published:** 2021-12-05

**Authors:** Vivian Caso Coelho, Samara D’Ajuda Pereira Neves, Mauro Cintra Giudice, Gil Benard, Marta Heloisa Lopes, Paula Keiko Sato

**Affiliations:** 1 Laboratory of Medical Investigation in Immunology (LIM48), Hospital das Clinicas HCFMUSP, Faculdade de Medicina, Universidade de Sao Paulo, Av. Dr. Eneas Carvalho de Aguiar 470, IMT 2, terreo, Sao Paulo, SP 05403-000 Brazil; 2 Department of Infectious Diseases, FMUSP, Universidade de Sao Paulo, Sao Paulo, Brazil; 3 Programa de Aprimoramento Profissional, HCFMUSP (LIM48), Sao Paulo, Brazil; 4 Laboratory of Medical Investigation in Micology (LIM53), HCFMUSP, Sao Paulo, Brazil; 5 Instituto de Medicina Tropical de Sao Paulo, Faculdade de Medicina FMUSP, Universidade de Sao Paulo, Sao Paulo, Brazil; 6 Present Address: Faculdades Oswaldo Cruz, Sao Paulo, Brazil

**Keywords:** Nocardia, Antimicrobial susceptibility testing, Broth microdilution, Resazurin, Spectrophotometry, Minimum inhibitory concentration

## Abstract

**Background:**

*Nocardia* species are ubiquitous in natural environments and can cause nocardiosis. In the present study, the use of Resazurin salt and Spectrophotometry were proposed as alternative methods to reduce subjectivity in the interpretation of susceptibility results to antimicrobials by the broth microdilution method for *Nocardia* spp.

**Results:**

The susceptibility of *Nocardia* spp. isolates to Amikacin, Ciprofloxacin, Minocycline and Trimethoprim-Sulfamethoxazole was evaluated by Minimum Inhibitory Concentration (MIC) determinations by the broth microdilution method. To verify cellular growth, the colour-changing dye Resazurin was applied, the Optical Densities were measured on a spectrophotometer, and both were compared to Clinical and Laboratory Standards Institute (CLSI) Gold Standard method (visual MIC determination). Percentages of essential and categorical agreements and interpretative categorical errors were calculated within each method (intra-reading) and between them (inter-reading). The Gold Standard visual reading demonstrated 100% of essential and categorical intra-reading agreements for Amikacin, and there was no error when compared with the alternative methods. For Ciprofloxacin, the comparison between the Gold Standard and the Spectrophotometric reading showed 91.5% of essential agreement. In the categorical intra-reading analysis for Minocycline, there were 88.1 and 91.7% in the Gold Standard and in the Spectrophotometric readings, respectively, and 86.4% of concordance between them. High rates of categorical agreement were also observed on the Trimethoprim-Sulfamethoxazole analyses, with 93.7% for the Gold Standard, 84.9% for the Resazurin readings, and 80.5% between them.

**Conclusions:**

The alternative methods with Resazurin and Spectrophotometric readings showed high agreement rates with the Gold Standard.

## Background

The genus *Nocardia* sp. is a large group of gram-positive bacteria, aerobically living in soil, organic matter, and fresh or salt water. *Nocardia* belong to the family *Nocardiaceae*, from the suborder of aerobic actinomycetes that also includes *Mycobacterium*, *Corynebacterium*, *Gordona* and *Tsukamurella* [1, 2].

Several species of *Nocardia* can cause disease in humans, with a wide spectrum of clinical manifestations. In immunocompetent individuals, nocardiosis commonly occurs after traumatic inoculation of the microorganism, while the respiratory tract is often the initial site of infection in the immunocompromised [3]. Despite being potentially fatal as an opportunistic disease, the prevalence of nocardiosis is unknown in most countries, as it is not compulsorily notified. Additionally, the identification of *Nocardia* is challenging. These bacteria grow in specific media for mycobacteria and form partially acid-fast beaded branching filaments, similarly to mycobacteria, which frequently results in *Nocardia* being mistaken for *Mycobacterium* [4].

The identification of the species is necessary for an adequate treatment, but its effectiveness may depend on the antimicrobial susceptibility profile, widely diverse among *Nocardia* spp. Since nocardiosis can be fatal in the absence of treatment and there were reports of *Nocardia* resistance to sulfonamides, the susceptibility testing becomes even more important. In addition, there are new antibiotic agents that could be even more effective than the usual sulfonamide treatment [5, 6].

The Clinical and Laboratory Standards Institute (CLSI) currently recommends the Broth Microdilution (BMD) method to determine the Minimum Inhibitory Concentrations (MIC) of antimicrobial agents to establish the susceptibility of *Nocardia* spp. isolates [7]. However, Brown-Elliott et al. [8] already addressed the subjectivity in the visualization and interpretation of BMD method results. As an effort to reduce this bias, some reports applied the Resazurin, a blue reagent that changes to pink when reduced to resorufin because of the growth of cultured cells, such as bacteria and fungi [9–11]. Likewise, spectrophotometric readings of Optical Densities (OD) were also used to diminish subjective interpretation of BMD method results [12–14].

In the present study, the application of Resazurin and OD readings were investigated as alternative methods that could reduce subjectivity in the interpretation of susceptibility to antimicrobials testing on *Nocardia* spp. by broth microdilution assay.

## Results

The *Nocardia* spp. isolates were evaluated in this study by the BMD method, and the use of Resazurin salt (visually determined MIC with Resazurin) and the OD reading (spectrophotometric determined MIC by ODs) were evaluated as alternative methods of result reading in comparison with the Gold Standard visual reading (CLSI recommended method with visually determined MIC).

The susceptibility profile of the evaluated *Nocardia* isolates, with respective modal MIC and ranges for Amikacin (AMK), Ciprofloxacin (CIP), Minocycline (MIN), and Trimethoprim-Sulfamethoxazole (TMP-SMX) by BMD method are presented on Table 1. Fifteen isolates were analyzed and most were susceptible to the evaluated antimicrobial drugs; four and ten isolates were resistant to MIN and to CIP, respectively.

**Table.1:** Reading methods results of MIC and susceptibility from broth microdilution assays for *Nocardia* spp. isolates

Isolates	Drugs	Visual Readings				OD Readings	
		Gold Standard		With Resazurin			
		Modal MIC (range)	^a^Susc. (%)	Modal MIC (range)	^a^Susc. (%)	Modal MIC (range)	^a^Susc. (%)
*N. brasiliensis*-83	AMK	0.5 (0.5–2.0)	100.0	0.5 (0.5–1.0)	100.0	1.0 (0.5–1.0)	100.0
	CIP	0.12 (0.12)	100.0	0.12 (0.12)	100.0	0.12 (0.12)	100.0
	MIN	8.0 (4.0–16.0)	0.0	4.0 (4.0–8.0)	0.0	4.0 (2.0–8.0)	0.0
	TMP–SMX	0.5 (0.12–2.0)	100.0	4.0 (0.25–8.0)	37.5	0.25 (0.12–0.5)	100.0
*N. brasiliensis*-95	AMK	0.5 (0.5–1.0)	100.0	2.0 (0.5–2.0)	100.0	1.0 (0.5–1.0)	100.0
	CIP	4.0 (4.0)	0.0	4.0 (4.0)	0.0	4.0 (2.0–4.0)	0.0
	MIN	1.0 (0.25–1.0)	100.0	0.5 (0.25–2)	90.0	1.0 (0.5–1.0)	100.0
	TMP–SMX	0.12 (< 0.12–0.5)	100.0	0.12 (0.12–2)	100.0	0.12 (< 0.12–2.0)	100.0
*N. brasiliensis*-129	AMK	1.0 (0.5–1.0)	100.0	2.0 (2.0–4.0)	100.0	1.0 (1.0–2.0)	100.0
	CIP	4.0 (4.0)	0.0	4.0 (4.0)	0.0	4.0 (2.0–4.0)	0.0
	MIN	0.5 (0.25–1.0)	100.0	1.0 (0.25–1.0)	100.0	0.5 (0.25–1.0)	100.0
	TMP–SMX	0.25 (0.12–0.5)	100.0	0.25 (0.25–1.0)	100.0	0.25 (0.25–1.0)	100.0
*N. brasiliensis*-514	AMK	0.5 (0.5–1.0)	100.0	0.5 (0.5–1.0)	100.0	0.5 (0.5–4.0)	100.0
	CIP	0.12 (0.12)	100.0	0.12 (0.12)	100.0	0.12 (0.12)	100.0
	MIN	4.0 (4.0–16.0)	0.0	8.0 (4.0–8.0)	0.0	4.0 (2.0–8.0)	0.0
	TMP–SMX	2.0 (0.25–8.0)	62.5	4.0 (2.0–32.0)	14.3	0.12 (0.12–8.0)	87.5
*N. brasiliensis*-519	AMK	0.5 (0.5)	100.0	0.5 (0.5–1.0)	100.0	1.0 (0.5–1.0)	100.0
	CIP	2.0 (1.0–2.0)	50.0	1.0 (1.0–2.0)	71.4	2.0 (2.0)	0.0
	MIN	0.5 (0.06–1.0)	100.0	1.0 (0.12–2.0)	80.0	0.25 (0.06–0.25)	100.0
	TMP–SMX	0.12 (0.12–0.25)	100.0	0.12 (0.12–1.0)	100.0	0.12 (0.12–0.25)	100.0
*N. brasiliensis*-608	AMK	2.0 (0.5–2.0)	100.0	2.0 (0.5–2.0)	100.0	2.0 (2.0)	100.0
	CIP	4.0 (0.12–32.0)	22.2	4.0 (2.0–4.0)	0.0	4.0 (0.12–4.0)	33.3
	MIN	0.06 (0.06–0.12)	100.0	0.06 (0.06–0.5)	100.0	0.06 (0.06–0.12)	100.0
	TMP–SMX	1.0 (0.25–1.0)	100.0	0.5 (0.12–1.0)	100.0	0.25 (0.12–025)	100.0
*N. brasiliensis*-775	AMK	0.5 (0.5–1.0)	100.0	0.5 (0.5–1.0)	100.0	1.0 (0.5–1.0)	100.0
	CIP	4.0 (4.0)	0.0	4.0 (4.0)	0.0	4.0 (4.0)	0.0
	MIN	0.06 (0.06–0.5)	100.0	1.0 (0.06–1.0)	100.0	0.06 (0.06–0.25)	100.0
	TMP–SMX	1.0 (0.12–1.0)	100.0	1.0 (0.25–4.0)	66.7	0.25 (0.25–1.0)	100.0
*N. brasiliensis*-865	AMK	1.0 (0.5–2.0)	100.0	2.0 (1.0–2.0)	100.0	0.5 (0.5–2.0)	100.0
	CIP	4.0 (0.25–8.0)	11.1	4.0 (2.0–4.0)	0.0	4.0 (2.0–4.0)	0.0
	MIN	0.5 (0.5–1.0)	100.0	0.12 (0.06–0.12)	100.0	1.0 (0.5–1.0)	100.0
	TMP–SMX	0.12 (0.12–0.25)	100.0	0.12 (0.12)	100.0	0.12 (0.12–0.25)	100.0
*N. farcinica*-935	AMK	0.5 (0.5–1.0)	100.0	1.0 (0.5–2.0)	100.0	0.5 (0.5–1.0)	100.0
	CIP	64.0 (32.0–64.0)	0.0	64.0 (32.0–64.0)	0.0	16.0 (16.0–32.0)	0.0
	MIN	0.12 (0.06–1.0)	100.0	0.06 (0.06–2.0)	91.6	0.06 (0.06–0.12)	100.0
	TMP–SMX	1.0 (0.25–4.0)	83.3	4.0 (0.25–8.0)	41.6	0.25 (0.25–1.0)	100.0
*N. otitidiscaviarum*-779	AMK	0.5 (0.5–1.0)	100.0	0.5 (0.5–1.0)	100.0	0.5 (0.5–1.0)	100.0
	CIP	4.0 (4.0–64.0)	0.0	8.0 (8.0–16.0)	0.0	8.0 (1.0–4.0)	33.3
	MIN	0.06 (0.06–0.25)	100.0	0.06 (0.06–1.0)	100.0	0.06 (0.06–0.25)	100.0
	TMP–SMX	0.5 (0.25–0.5)	100.0	0.5 (0.25–8.0)	62.5	0.5 (0.12–1.0)	100.0
*N. asteroides*-788	AMK	0.5 (0.5–1.0)	100.0	1.0 (0.5–2.0)	100.0	0.5 (0.5)	100.0
	CIP	0.5 (0.25–2.0)	100.0	0.5 (0.5–2.0)	100.0	0.25 (0.25–1.0)	100.0
	MIN	4.0 (2.0–4.0)	0.0	4.0 (4.0–8.0)	0.0	2.0 (2.0–4.0)	0.0
	TMP–SMX	2.0 (0.25–4.0)	75.0	2.0 (0.25–32.0)	50	0.25 (0.12–0.5)	100.0
*N. otitidiscaviarum*-937	AMK	0.5 (0.5–1.0)	100.0	0.5–1.0 (1.0)	100.0	0.5 (0.5–1.0)	100.0
	CIP	2.0 (1.0–2.0)	22.2	2.0 (2.0–4.0)	0.0	2.0 (0.25 –2.0)	28.6
	MIN	0.06 (0.06–0.12)	100.0	0.06 (0.06–1.0)	100.0	0.06 (0.06–0.25)	100.0
	TMP–SMX	1.0 (0.25–1.0)	100.0	1.0 (0.25–2.0)	100.0	0.25 (0.12–0.5)	100.0
*N. beijingensis*-1180	AMK	0.5 (0.5–1.0)	100.0	0.5 (0.5–1.0)	100.0	0.5 (0.5)	100.0
	CIP	2.0 (2.0–4.0)	0.0	4.0 (2.0–4.0)	0.0	2.0 (2.0–8.0)	0.0
	MIN	1.0 (0.5–2.0)	60.0	1.0 (1.0–2.0)	90.0	2.0 (1.0–4.0)	30.0
	TMP–SMX	0.12 (0.12–0.25)	100.0	0.12 (0.12–0.25)	100.0	0.12 (0.12–1.0)	100.0
*N. beijingensis*-1181	AMK	0.5 (0.5–1.0)	100.0	0.5 (0.5–1.0)	100.0	0.5 (0.5–1.0)	100.0
	CIP	4.0 (1.0–4.0)	9.1	4.0 (0.12–8.0)	20.0	4.0 (2.0–4.0)	0.0
	MIN	1.0 (0.5–2.0)	60.0	1.0 (0.5–2.0)	66.6	2.0 (1.0–4.0)	20.0
	TMP–SMX	0.12 (0.12–0.25)	100.0	0.12 (0.12–0.25)	100.0	0.12 (0.12–0.5)	100.0
*N. farcinica*-2901	AMK	1.0 (1.0–2.0)	100.0	2.0 (2.0–4.0)	100.0	1.0 (1.0)	100.0
	CIP	0.25 (0.25)	100.0	0.5 (0.25–4.0)	85.7	0.25 (0.25)	100.0
	MIN	2.0 (2.0)	0.0	2.0 (2.0)	0.0	2.0 (1.0–2.0)	50.0
	TMP–SMX	8.0 (4.0–8.0)	0.0	16.0 (16.0)	0.0	1.0 (0.5–1.0)	100.0

^a^Percentage of Susceptibility

The percentages of Essential (MIC values) and Categorical (susceptibility classification) Agreements were calculated for intra- (same reading method, independent assays) and inter-reading (different reading methods, same assay) comparisons, considering ±1 two-fold dilution for concordant results. The Pearson Correlation Coefficients (Pearson’s r) and respective 95% Confidence Intervals (95% CI) were determined on the inter-reading methods comparisons of MIC values. The Pearson’s r-values range from - 1 to + 1: values from 0 to + 1 indicate a positive correlation, from 0 to - 1 indicate a negative correlation, and 0.0 indicates no correlation at all. *P*-values of < 0.05 indicate a significant correlation (Table 2).

**Table 2:** Agreements (%) on comparisons within and between reading methods for BMD of *Nocardia* spp. isolates

Drugs	Agreement		Gold Standard	Resazurin	OD
**AMK**	Essential	Intra-reading	98.4	97.6	98.3
		^a^Inter-reading	―	90.8	99.2
		^a,b^Pearson’s r (95% CI)	―	0.50 (0.36–0.63)	0.92 (0.88–0.94)
	Categorical	Intra-reading	100.0	100.0	100.0
		^a^Inter-reading	―	100.0	100.0
		^a^Errors: Very Major	―	0.0	0.0
		Major	―	0.0	0.0
		Minor	―	^c^n.a.	^c^n.a.
**CIP**	Essential	Intra-reading	91.7	94.4	93.4
		^a^Inter-reading	―	90.7	89.4
		^a,b^Pearson’s r (95% CI)	―	0.69 (0.58–0.77)	0.82 (0.76–0.87)
	Categorical	Intra-reading	82.7	88.1	77.9
		^a^Inter-reading	―	73.7	75.0
		^a^Errors: Very Major	―	0.85	3.79
		Major	―	4.24	1.52
		Minor	―	21.2	19.7
**MIN**	Essential	Intra-reading	87.2	78.8	91.5
		^a^Inter-reading	―	80.0	93.8
		^a,b^Pearson’s r (95% CI)	―	0.90 (0.86–0.92)	0.90 (0.87–0.93)
	Categorical	Intra	88.0	89.8	91.5
		^a^Inter	―	85.6	87.7
		^a^Errors: Very Major	―	0.0	0.0
		Major	―	0.0	0.0
		Minor	―	14.4	12.3

^a^Compared with the Gold Standard

^b^*P* < 0.0001 for all comparisons

^c^Not applicable

The percentages of Essential Agreement (EA) for the three drugs on both intra- and inter-reading comparisons were higher than 80.0%, and there were positive and significant correlations between the Gold Standard and both alternative methods, Resazurin and OD readings (*P* < 0.0001 for all comparisons). The EA percentages for CIP assays between the Gold Standard and the Resazurin readings, and between the Gold Standard and the OD readings were 90.7 and 89.4%, respectively. For the AMK assays, all three reading methods showed 100% of Categorical Agreement (CA) in both intra- and inter-reading comparisons (Table 2).

For the CA, discrepancies of classification were calculated as percentages of errors defined as: Minor error, an intermediate result in the alternative assay and a resistant or susceptible result in the Gold Standard method, or vice-versa; Major error, a susceptible result in the Gold Standard method, but resistant in the alternative assay (false resistance); and Very Major error, a resistant result in the Gold Standard method, but susceptible in the alternative assay (false susceptible). Acceptable error levels were ≤ 10% for Minor, ≤ 3.0% for Major, and ≤ 1.5% for Very Major errors [14].

There were no categorical errors on the results of AMK. For CIP, on the other hand, 0.85% of Very Major errors were observed between the Gold Standard and the Resazurin readings, and 1.52% of Major errors between the Gold Standard and the OD readings. For the MIN assays, there were no Very Major and Major errors in the inter-reading comparisons, and the percentage of Minor errors ranged from 12.3 to 14.4% (Table 2).

The Disk Diffusion test is recommended by the CLSI [7] to confirm MIC results from *Nocardia* spp. isolates with breakpoints at the limit between antimicrobial susceptibility and resistance, for there is no classification of intermediate resistance for TMP-SMX. Forty-four assays were performed with *Nocardia* spp. isolates and all were susceptible to this drug. In addition, the inter-reading comparison between the Gold Standard and the Disk Diffusion tests showed 97.7% of Categorical Agreement, and 2.27% of Very Major errors (Table 3).

**Table.3:** Agreements within and between susceptibility assays of *Nocardia* spp. isolates with TMP-SMX

Agreement	^**a**^Broth Microdilution Method						Disk Diffusion
	Gold Standard		Resazurin		OD		
	± 1	± 2	± 1	± 2	± 1	± 2	
**Essential**							
Intra-reading	82.0	97.0	73.1	88.5	81.8	93.9	^d^n.a.
^b^Inter-reading	―	―	71.7	89.8	75.0	88.6	^d^n.a.
^b,c^Pearson’s r	―	―	0.50	0.42	0.36	0.44	^d^n.a.
(95% CI)			(0.36–0.62)	(0.26–0.55)	(0.20–0.45)	(0.30–0.57)	
**Categorical**							
Intra-reading	95.5	88.5	86.2	86.2	99.2	97.0	100.0
^b^Inter-reading	―	―	81.1	91.3	91.7	93.9	97.7
^b^Very Major Errors	―	―	0.0	0.0	8.33	6.06	2.27
^b^Major Errors	―	―	18.9	8.66	0.0	0.0	0.0

^a^BMD method concordant results considering ±1 or ± 2 two-fold dilutions

^b^Compared with the Gold Standard BMD method

^c^*P* < 0.0001 for all comparisons

^d^Not applicable

For TMP-SMX, the Essential Agreements, the Pearson’s r-values with 95% CI, the Categorical Agreements and Errors were calculated for the intra- and inter-reading methods comparisons considering ±1 and ± 2 two-fold dilutions for concordant results. The percentages of EA on both intra- and inter-reading comparisons increased when ±2 two-fold dilutions were considered, nevertheless, the Pearson’s r-values were significant on all comparisons (*P* < 0.0001). The inter-reading comparison between the Gold Standard and the OD readings showed 91.7% of CA with ±1 two-fold and 93.9% with ±2 two-fold dilutions. Despite of CA being greater than 90%, the percentage of Very Major errors for ±1 two-fold and ± 2 two-fold dilutions were 8.33 and 6.06%, respectively (Table 3).

## Discussion

The broth microdilution test determines the MIC of an antimicrobial agent, however, the interpretation of results is still challenging for isolates of *Nocardia* spp. The results are visually determined, and therefore, subjective and more prone to errors by laboratory technicians [15].

If there is doubt about the MIC results of TMP-SMX by the broth microdilution method, CLSI recommends the Disk Diffusion test to be performed [7]. In fact, we found difficult to determine the susceptibility classification of the *N. brasiliensis*-514 isolate by the broth microdilution method, with MICs varying between susceptible and resistant ranges. Hence, the Disk Diffusion test was employed and it was easy to read and to determine this isolate as susceptible for TMP-SMX.

The Resazurin readings in this study determined the MIC after 72 h of incubation, and resulted on the resistance to CIP of *N. otitidiscaviarum* and *N. beijingensis*, as well as the susceptibility to AMK and TMP-SMX of all isolates. These data are similar to that previously reported on *Nocardia* spp. isolates and the use of Alamar Blue [16], which is a commercial dye solution of Resazurin [17]. We also showed high percentages of Categorical Agreement between the Gold Standard and the Resazurin readings (71.7–100%), corroborating with the statement from Zhao et al. that the test is cheap and reliable and its application in *Nocardia* sp. could improve the international standardization of susceptibility testing methods [16].

The comparisons between the Gold Standard and the OD readings showed high percentages of Essential (75.0–99.2%) and Categorical (75.0–100%) Agreements. Similarly, Meletiadis et al. demonstrated that the European Committee on Antimicrobial Susceptibility Testing (EUCAST) spectrophotometric MIC determination for antifungal susceptibility testing with *Aspergillus* spp. isolates was in excellent Essential (92–97%) and Categorical (93–99%) Agreements with the visual MIC determination [14].

To the best of our knowledge, there are no studies using Spectrophotometry (OD readings) for *Nocardia* spp., but our results demonstrate its applicability for these bacteria, as it is currently used for fungal agents.

EUCAST, unlike CLSI, does not have a specific recommendation or defined breakpoints for *Nocardia* sp., however, their documents are free of charge and easily accessible to clinical laboratories for other microorganisms. CLSI documents, on the other hand, are only available through a costly payment, which can restrict their access and hinder their use by emerging countries. We showed that the use of Resazurin and Spectrophotometry could help defining a novel document and breakpoints for *Nocardia* spp. isolates.

The small number of *Nocardia* spp. isolates and evaluated drugs were the main limitations of this study. Nevertheless, the results herein disclosed the promising use of Resazurin and OD readings in future investigations on antimicrobial susceptibility of *Nocardia* spp. isolates.

## Conclusions

Our data suggest that Resazurin and Spectrophotometry are efficient, reproducible and easy-to-use alternative methods for determining the susceptibility profile of *Nocardia* spp.

## Methods

### *Nocardia* spp. isolates and ATCC strain

*Nocardia* spp. isolates from the Collection of Cultures of the Laboratory of Medical Research in Mycology (LIM53) were included in this study (*n* = 15; Table 1). Eight isolates were identified in a previous study [4] and the other seven *Nocardia beijingensis*(*N. beijingensis*)-1180 (MW348983), *N. beijingensis*-1181 (MW348984), *Nocardia brasiliensis*(*N. brasiliensis*)-95 (MW348931), *N. brasiliensis*-129 (MW348933), *N. brasiliensis*-519 (MW348935), *Nocardia farcinica*(*N. farcinica*)-788 (MW348936), and *N. farcinica*-2901 (MW348955) were identified by Gram and Ziehl Neelsen staining methods, morphological characteristics and molecular sequencing. A 606 bp fragment of the *16S* rRNA gene was amplified with the primers *Noc1* (5′-GCTTAACACATGCAAGTCG-3′) and *Noc2* (5′-GAATTCCAGTCTCCCCTG-3′), and sequenced as previously described [18]. The species was confirmed by comparing them against type strain sequences with the BLAST algorithm v.2.2.10 (http://www.ncbi.nlm.nih.gov/BLAST). Similarity values of ≥98.0% for *16S* rRNA were deemed to indicate the same species.

CLSI-recommended quality control ranges for Amikacin (AMK), Ciprofloxacin (CIP), Minocycline (MIN) and Trimethoprim-Sulfamethoxazole (TMP-SMX) were performed by using a strain of *Staphylococcus aureus* (*S. aureus* ATCC 29213) [7]. *Nocardia* spp. isolates were cultured on Tryptone Soy Agar (TSA; Kasvi, Italy) at 35 °C for 7 days and the ATCC strain was also cultured on TSA at 35 °C for 24 h.

### Resazurin solution

The 0.01% Resazurin solution was prepared by dissolving the powder dye (Sigma-Aldrich, Munich, Germany) in 1× PBS, pH 7.2 (Life Technologies, Carlsbad, CA, USA). After complete dissolution, the solution was sterilized by a 0.22 μm membrane filtration, stored at 4 °C, protected from light, and used within 3 days.

### Antimicrobial solutions

The antimicrobial solutions were prepared as recommended by the CLSI document M100-S24 [19]. Briefly, each antimicrobial agent (all from Sigma-Aldrich, Munich, Germany) was dissolved and diluted separately: AMK and MIN were dissolved and diluted in water; CIP was dissolved in Hydrochloric Acid 0.1 N and diluted in water; TMP was dissolved in Lactic Acid 0.05 M and diluted in warm water; and SMX was dissolved in Sodium Hydroxide 2.5 M and diluted in water. The drugs were two-fold serially diluted in CAMHB in 96-well microplates (100 μL/well; flat-bottomed cell culture microplates from Corning, Durham, NC, USA) at the following concentration ranges: 0.5–256.0 mg/L for AMK, 0.12–64.0 mg/L for CIP, 0.06–32.0 mg/L for MIN, and 0.12–64.0/2.37–1216.0 mg/L for TMP-SMX. The microplates were frozen at − 80 °C until use.

### Antimicrobial susceptibility test

The MICs were determined by the BMD method, according to CLSI document M24 for *Nocardia* [7]. Briefly, inoculum suspensions for each *Nocardia* isolate were prepared by dissolving the bacterial content with saline and glass microbeads (Sigma Aldrich, Munich, Germany), vortexing vigorously and then allowing it to precipitate for 10 to 15 min. The concentration was adjusted by dilution on Cation-Adjusted Mueller-Hinton Broth (CAMHB; Becton Dickinson and Company, Sparks, MD, USA).

Medium control wells had 200 μL/well of drug-free CAMHB, and inoculum control wells had 100 μL/well of drug-free CAMHB and 100 μL/well of bacterial inoculum. All the other wells were inoculated with 100 μL/well of bacterial inoculum on microplates with antimicrobial solutions, previously prepared. Microplates were incubated in a moist chamber at 35 °C.

Following incubation of 24 h, 20 μL/well of 0.01% sterile resazurin solution were added to all wells and microplates were re-incubated for 48 h for colour development. A change in colour from blue to pink indicated bacterial growth.

The ATCC strain inoculum suspension was prepared as described for *Nocardia* isolates, except for the incubation period. After incubation for 17 h, 20 μL/well of 0.01% sterile resazurin solution were added to all wells and the microplates were re-incubated for more 3 h for colour development.

Following the stablished incubation periods for the ATCC strain (17 and 20 h) and for *Nocardia* isolates (48 and 72 h), the microplates were evaluated by the Gold Standard visual reading without any dye, the visual reading with Resazurin, or by OD readings without any dye, at 560 nm in the Versa Max Microplate Reader spectrophotometer (Molecular Devices, Sunnyvale, CA, USA).

### Quality control

Growth in medium control wells indicated contamination or absence of growth in inoculum control wells indicated unviability of the isolate, and the corresponding assay was discarded. At least two wells were matched among the triplicates for a valid result.

### Disk diffusion test

The Disk Diffusion test was performed with the isolates suspensions, prepared as described on M24 CLSI document for *Nocardia* [7], and plated on 90-mm-diameter Mueller-Hinton agar plates (DME, Araçatuba, SP, Brazil). Antimicrobial disks of TMP-SMX (25.0 μg; Sensidisc, DME, Araçatuba, SP, Brazil) were then placed on the agar, and the plates were incubated at 35 °C for 3 to 5 days. Interpretive categories of Disk Diffusion results for *Nocardia* spp. isolates were based on the M24 CLSI document: a zone of ≥35 mm indicated susceptibility, and a zone of ≤15 mm indicated resistance.

### Analysis

For the visual interpretation of the results, the MIC well was determined by its comparison with medium and inoculum drug-free control wells. The MIC well was the one with the lowest drug concentration that prevented the bacterial growth and the colour change within the incubation period, being similar to the medium control well (no visible growth and blue with Resazurin). The inoculum with drug and the inoculum control drug-free wells should show visible growth and should be pink with resorufin. For the spectrophotometric readings, the percentages of bacterial growth were calculated with the following eq. [20]: bacterial growth percentage (%) = (OD from the inoculum with drug well - the OD from the medium control well) / (OD from the inoculum control drug-free well - OD the OD from the medium control well) × 100. The MIC well was the one with the lowest drug concentration with ≤10% of bacterial growth [14].

The Pearson Correlation Coefficients (Pearson’s r) and respective 95% Confidence Intervals (95% CI) were determined by the Prism 5.01 statistical software (GraphPad, San Diego, CA, USA).

## Data Availability

The datasets used and/or analysed during the current study are available from the corresponding author on reasonable request.

## Abbreviations

BMD: Broth Microdilution
AMK: Amikacin
CIP: Ciprofloxacin
MIN: Minocycline
TMP-SMX: Trimethoprim-Sulfamethoxazole
MIC: Minimum Inhibitory Concentration
OD: Optical Densities
CA: Categorical Agreement
EA: Essential Agreement

## References

[1] Wilson JW. Nocardiosis: updates and clinical overview. Mayo Clin Proc. 2012. 10.1016/j.mayocp.2011.11.016. 10.1016/j.mayocp.2011.11.016 PMC3498414 22469352

[2] Beaman BL, Burnside J, Edwards B, Causey W. Nocardial infections in the United States, 1972-1974. J Infect Dis. 1976. 10.1093/infdis/134.3.286. 10.1093/infdis/134.3.286 789786

[3] McNeil MM, Brown JM. The medically important aerobic actinomycetes: epidemiology and microbiology. Clin Microbiol Rev. 1994. 10.1128/CMR.7.3.357. 10.1128/cmr.7.3.357 PMC358331 7923055

[4] Muricy EC, Lemes RA, Bombarda S, Ferrazoli L, Chimara E. Differentiation between Nocardia spp. and Mycobacterium spp.: critical aspects for bacteriological diagnosis. Rev Inst Med Trop Sao Paulo. 2014. 10.1590/s0036-46652014000500005. 10.1590/S0036-46652014000500005 PMC4172110 25229219

[5] Bibi S, Irfan S, Zafar A, Khan E. Isolation frequency and susceptibility patterns of Nocardia species at a tertiary hospital laboratory in Karachi, Pakistan. J Infect Dev Ctries. 2011. 10.3855/jidc.1278. 10.3855/jidc.1278 21727653

[6] Condas LA, Ribeiro MG, Muro MD, de Vargas AP, Matsuzawa T, Yazawa K, et al. Molecular identification and antimicrobial resistance pattern of seven clinical isolates of Nocardia spp. in Brazil. Rev Inst Med Trop Sao Paulo. 2015. 10.1590/S0036-46652015000300012. 10.1590/S0036-46652015000300012 PMC4544251 26200967

[7] Clinical and Laboratory Standards Institute (2018). Susceptibility testing of mycobacteria, Nocardiae, and other aerobic actinomycetes. Approved standard, 3rd ed. CLSI Document M24.

[8] Brown-Elliott BA, Brown JM, Conville PS, Wallace RJ Jr. Clinical and laboratory features of the Nocardia spp. based on current molecular taxonomy. Clin Microbiol Rev. 2006. 10.1128/CMR.19.2.259-282.2006. 10.1128/CMR.19.2.259-282.2006 PMC1471991 16614249

[9] Palomino JC, Martin A, Camacho M, Guerra H, Swings J, Portaels F. Resazurin microtiter assay plate: simple and inexpensive method for detection of drug resistance in Mycobacterium tuberculosis. Antimicrob Agents Chemother. 2002. 10.1128/aac.46.8.2720-2722.2002. 10.1128/AAC.46.8.2720-2722.2002 PMC127336 12121966

[10] Castilho AL, Caleffi-Ferracioli KR, Canezin PH, Dias Siqueira VL, de Lima Scodro RB, Cardoso RF. Detection of drug susceptibility in rapidly growing mycobacteria by resazurin broth microdilution assay. J Microbiol Methods. 2015. 10.1016/j.mimet.2015.02.007. 10.1016/j.mimet.2015.02.007 25683207

[11] de Paula e Silva AC, Oliveira HC, Silva JF, Sangalli-Leite F, Scorzoni L, Fusco-Almeida AM, et al. Microplate alamarBlue assay for Paracoccidioides susceptibility testing. J Clin Microbiol. 2013. 10.1128/JCM.02914-12. 10.1128/JCM.02914-12 PMC3666786 23345296

[12] Al-Hatmi AM, Normand AC, Ranque S, Piarroux R, de Hoog GS, Meletiadis J, et al. Comparative evaluation of Etest, EUCAST, and CLSI methods for amphotericin B, voriconazole, and posaconazole against clinically relevant Fusarium species. Antimicrob Agents Chemother. 2016. 10.1128/AAC.01671-16. 10.1128/AAC.01671-16 PMC5192122 27795379

[13] Foerster S, Desilvestro V, Hathaway LJ, Althaus CL, Unemo M. A new rapid resazurin-based microdilution assay for antimicrobial susceptibility testing of Neisseria gonorrhoeae. J Antimicrob Chemother. 2017. 10.1093/jac/dkx113. 10.1093/jac/dkx113 PMC5890744 28431096

[14] Meletiadis J, Leth Mortensen K, Verweij PE, Mouton JW, Arendrup MC. Spectrophotometric reading of EUCAST antifungal susceptibility testing of Aspergillus fumigatus. Clin Microbiol Infect. 2017. 10.1016/j.cmi.2016.10.017. 10.1016/j.cmi.2016.10.017 27793736

[15] Conville PS, Brown-Elliott BA, Smith T, Zelazny AM. The complexities of Nocardia taxonomy and identification. J Clin Microbiol. 2018. 10.1128/JCM.01419-17. 10.1128/JCM.01419-17 PMC5744224 29118169

[16] Zhao P, Zhang X, Du P, Li G, Li L, Li Z. Susceptibility profiles of Nocardia spp. to antimicrobial and antituberculotic agents detected by a microplate Alamar Blue assay. Sci Rep. 2017. 10.1038/srep43660. 10.1038/srep43660 PMC5333629 28252662

[17] O'Brien J, Wilson I, Orton T, Pognan F (2000). Investigation of the Alamar Blue (resazurin) fluorescent dye for the assessment of mammalian cell cytotoxicity. Eur J Biochem.

[18] Rodríguez-Nava V, Couble A, Devulder G, Flandrois JP, Boiron P, Laurent F. Use of PCR-restriction enzyme pattern analysis and sequencing database for hsp65 gene-based identification of Nocardia species. J Clin Microbiol. 2006. 10.1128/JCM.44.2.536-546.2006. 10.1128/JCM.44.2.536-546.2006 PMC1392680 16455910

[19] Clinical and Laboratory Standards Institute (2014). Performance standards for antimicrobial susceptibility testing: informational supplement. CLSI document M100-S24.

[20] Liu M, Seidel V, Katerere DR, Gray AI. Colorimetric broth microdilution method for the antifungal screening of plant extracts against yeasts. Methods. 2007. 10.1016/j.ymeth.2007.02.013. 10.1016/j.ymeth.2007.02.013 17560320

